# Dietary Encapsulated Olive-Derived Polyphenols: Productive Performance and Meat Quality in Podolian Young Bulls

**DOI:** 10.3390/ani16121791

**Published:** 2026-06-10

**Authors:** Dereje Birara Teshale, Siria Tavaniello, Marisa Palazzo, Meng Peng, Giulia Grassi, Innocenzo Muzzalupo, Giuseppe Maiorano

**Affiliations:** 1 Dipartimento di Agricoltura, Ambiente e Alimenti, Università degli Studi del Molise, 86100 Campobasso, Italy; d.teshale@studenti.unimol.it (D.B.T.); m.palazzo@unimol.it (M.P.); m.peng@studenti.unimol.it (M.P.); giulia.grassi@unimol.it (G.G.); maior@unimol.it (G.M.); 2 Consiglio per la Ricerca in Agricoltura e l’Analisi dell’Economia Agraria—Centro di Ricerca Foreste e Legno, 87036 Rende, Italy; innocenzo.muzzalupo@crea.gov.it

**Keywords:** beef cattle, olive leaves, olive mill wastewater, meat fatty acid composition, meat oxidative stability

## Abstract

This study evaluated the effects of dietary supplementation with nano- and micro-encapsulated polyphenol extract (PE) from olive leaves (OL) and olive mill wastewater (OMWW), respectively, on growth, carcass and meat quality traits in Podolian young bulls. Fifteen 12-month-old bulls were assigned to three groups: C (control); T1 (40 g/day nano-encapsulated PE from OL); and T2 (400 g/day olive leaf pellets plus 30 g/day micro-encapsulated PE from OMWW) for 40 days. Final body weight and carcass yield were unaffected, although the average daily gain was higher in T2 (*p* < 0.05). Meat from T2 exhibited lower moisture and higher protein content (*p* < 0.01) compared with the other groups. T1 showed higher α-tocopherol levels (*p* < 0.05). Lipid oxidation was reduced in both treated groups (*p* < 0.01). Monounsaturated fatty acids tended to decrease in treated groups (*p* = 0.057), while saturated and polyunsaturated fatty acids (PUFA) were unaffected. However, T2 showed higher total n-3 PUFA (*p* < 0.05), and a more favourable n-6/n-3 ratio (*p* < 0.01) was found in treated groups. These results highlight the potential of olive–derived polyphenols as functional feed ingredients to enhance meat quality and promote sustainable, circular livestock systems.

## Simple Summary

This study investigated the impact of adding natural compounds from olive oil processing by-products to the diet of Podolian young bulls. Two supplementation strategies for 40 days were used: one using nano-encapsulated polyphenol extracts from olive leaves, and another combining olive leaf pellets with micro-encapsulated extracts from olive mill wastewater. The results showed that the combination of olive leaf pellets and micro-encapsulated extracts improved the average daily weight gain of the bulls. The supplemented diets enhanced the nutritional profile of the meat by increasing protein content and vitamin E levels while significantly reducing lipid oxidation. Moreover, the combination of olive leaf pellets and micro-encapsulated extracts led to a more favorable meat nutritional profile by increasing the total levels of healthy n-3 fatty acids. This research supports the development of sustainable, circular livestock systems by transforming agricultural waste into valuable functional feed ingredients that enhance meat quality.

## 1. Introduction

Extensive livestock systems in marginal Mediterranean areas rely on rustic, locally adapted breeds that can convert low-quality forages into high-value animal products. Among these, Podolian cattle represent a key biological and socio-economic resource, functioning as an “energy broker” within the trophic chain by transforming fibrous vegetation into nutrient-dense meat and dairy products. Podolian cattle are considered direct descendants of ancient wild cattle and are renowned for their exceptional rusticity [1]. This breed can play a multifunctional role as a “biological sentinel” of the landscape, supporting socio-economic development of these marginal areas. Unlike cosmopolitan cattle breeds, which require high-energy diets and controlled environments to express their genetic potential, the Podolica breed thrives due to specific evolutionary adaptations, including structural robustness, high rusticity, and high resistance to the harsh semi-arid environments of Southern Italy. The breed is predominantly managed under extensive grazing systems, often with minimal shelter, and thrives on poor-quality forage while maintaining reproductive efficiency under challenging environmental conditions [2,3,4]. The extensive rearing system adopted for these indigenous animals relies on grazing on natural (spontaneous) pastures, including grasslands and wood pastures, and scrub, including olive leaves, especially in winter. This production model contributes to the preservation of rural landscapes and enhances the distinctive qualitative traits of products obtained by the Podolian breed, which are closely linked to feeding regimes and agroecological conditions [5].

Olive cultivation (*Olea europaea* L.) is a defining feature of Mediterranean agriculture and plays a central role in the economic, social, and cultural identity of countries such as Spain, Tunisia, Italy, Morocco, and Greece [6,7]. In recent years, the interest in olive oil production and consumption has expanded olive cultivation to regions and countries outside the Mediterranean Basin, such as Australia, China, India, and South America [8]. This expansion is largely driven by rising international demand for high-quality oils and an increasing consumer awareness of the various health benefits associated with olive oil consumption, which include antioxidant, anti-inflammatory, and cardioprotective effects [9,10,11]. This expansion has increased the production of olive-derived residues, such as pruning material, leaves, pomace, and, especially, olive mill wastewater (OMWW), which pose significant environmental concerns [12]. Current disposal practices, such as burning of olive pruning residues, contribute to air pollution and are increasingly restricted under European agricultural policies [13]. OMWW is produced in large quantities, approximately 2.5 L per liter of olive oil, and represents one of the most problematic residues of the olive oil industry. The production of OMWW ranges from 5400 to 9700 × 10^3^ ton worldwide, or up to 30,000 × 10^3^ ton for the Mediterranean region [14]. Due to the large quantities produced in short periods, OMWW is characterized by a high phytotoxicity and a high level of organic pollution associated with a significant environmental impact [14,15]. These environmental risks have prompted growing interest in developing sustainable strategies for the treatment and valorization of OMWW and other olive by-products. Rather than being viewed as waste, OMWW is increasingly considered a valuable source of bioactive molecules, especially if we consider polyphenols with recognized antioxidant, antimicrobial, and anti-inflammatory activities [16]. In line with circular economy principles, which emphasize waste reduction, resource reuse, and environmental regeneration, the integration of agro-industrial residues into livestock diets represents a sustainable strategy for improving animal health and product quality. Several studies have highlighted the nutritional potential of such by-products in livestock feeding, not only as alternative sources of energy and protein but also as functional ingredients capable of improving animal health and product quality [17,18,19,20]. In meat production, the inclusion of natural antioxidants in ruminant diets is proposed as an effective method to improve meat quality, particularly in terms of oxidative stability and fatty acid profile [21,22]. In particular, olive by-products are increasingly recognized for their potential to enhance animal health and product quality due to their richness in bioactive compounds. Olive leaves, in particular, are rich in tannins that act as effective anthelmintic agents, reducing the production of enteric gases like methane and transferring natural antioxidant substances such as polyphenols and vitamins A, C, and E (α-tocopherol) into muscle tissue [23,24]. However, the direct incorporation of these olive-derived by-products into ruminant diets presents several practical limitations, such as seasonal availability, heterogeneous chemical composition, high moisture content, limited storage stability, susceptibility of phenolic compounds to oxidation and degradation, and difficulties in standardizing the amount of bioactive compounds supplied to animals. To overcome these limitations, emerging encapsulation technologies, such as nano- and micro-encapsulation, offer an effective means of stabilizing olive polyphenols, enhancing their bioavailability, and ensuring controlled release within the gastrointestinal tract [25]. Therefore, the use of encapsulated olive polyphenolic extracts as dietary supplements may play an important role in promoting animal welfare, improving meat quality, and supporting environmentally responsible livestock production. In this context, the comparison between a nano-encapsulated olive leaf extract and a practical combination of olive leaf pellet plus microencapsulated olive mill wastewater extract was designed to evaluate two different valorization strategies: a technologically advanced and concentrated polyphenol delivery system versus a more farm-applicable approach based on locally available olive by-products. Both strategies were formulated to provide comparable total polyphenol levels, allowing the assessment of their effects independently of major differences in total polyphenol supply. Consequently, this study investigated the effects of these distinct dietary olive polyphenol strategies on growth performance and meat quality in Podolian young bulls.

## 2. Materials and Methods

The experimental procedures were evaluated and approved by the Ethical Committee of the University of Molise with authorization n. 13084 (21 March 2024) and carried out according to international guidelines on the protection of animals used for scientific research (Directive 2010/63/EU).

### 2.1. Animals and Experimental Design

The study was conducted in the early spring on a Podolian cattle farm located in the Calabria region (“La Sulla”, Rossano Calabro, Cosenza; Latitude: 39.573226, Longitude: 16.634398) in a hilly area at an altitude of approximately 500 m above sea level. For the trial, 15 Podolian young bulls (*Bos primigenius Taurus*) with an average age of 12 months, born in the same season, weaned and reared under the same management and feeding systems, were used. Animals were chosen with an initial live weight of approximately 290 kg. This average weight was chosen to enable animals to reach the target slaughter weight of approximately 330 kg within the trial duration, considering the planned experimental period of 40 days and the expected average daily gain consistent with the breed and feeding system adopted. The target slaughter weight was established to comply with market requirements and consumer preferences of the local catchment area served by the farm, located in Rossano Calabro, where the size of cuts and meat quality obtained by this live weight are commonly preferred by regional retailers and consumers. This strategy ensured that the experimental design reflected realistic production conditions and commercial standards. At the beginning of the trial, animals were individually identified and weighed. Then, bulls were randomly subdivided into three homogeneous groups based on the average body weight: control (C; *n* = 5; initial live weight 294.50 ± 29.23 kg) received a concentrate based on durum wheat flour, barley, and faba bean (4.0 kg), along with meadow hay (3.0 kg) supplied at about 2% of the initial live weight; T1 (*n* = 5; initial live weight 316.80 ± 21.34 kg) fed with the same diet administered to the control group, at about 2% of the initial live weight, supplemented with 40 g/day/head of nano-encapsulated polyphenolic extracts from OL; T2 (*n* = 5; initial live weight 278.67 ± 42.25 kg) fed with the same diet administered to the control group, at about 2% of the initial live weight, supplemented with 400 g/day/head of olive leaf pellets and 30 g/day/head of micro-encapsulated polyphenolic extract from OMWW (Supplementary Table S1). Each animal, fed individually, received the same amount of feed in the morning and afternoon since the daily feed ration was divided into two administrations and the offered amount was completely consumed by the animals. In the morning, once the feed was consumed, the animals had access to the pasture and returned to the barn. A tie stall system was adopted from the farm and each animal had its own manger. Water was available *ad libitum*. The pasture area, extending over approximately 20 ha, was characterized by a typical Mediterranean climate. Pasture sampling was performed using a hand-plucking method, collecting representative samples from different areas of the grazing paddock at the same time of day (mid-morning) to represent the forage effectively selected by the animals. The pasture was predominantly composed of grasses (approximately 55–60%), legumes (25–30%), and other spontaneous herbaceous species and shrubs (10–15%). The grass component was mainly represented by *Avena* spp. and *Bromus* spp., while the dominant legumes included *Medicago* spp. and *Trifolium* spp. The spontaneous shrub layer comprised species such as *Quercus ilex*, *Pistacia lentiscus*, and *Myrtus communis*. Chemical analyses of the pasture were carried out through two samplings of the available vegetation, performed at the middle and at the end of the 40-day experimental trial. Considering the short duration of the experimental period and the overall homogeneity of the pasture botanical composition, all the collected subsamples were subsequently pooled into a single composite sample, which was then dried and analyzed for chemical composition.

The ingredients and the chemical composition of the farm diet and pasture, determined according to the methods described by the Association of Official Analytical Chemists [26] and Van Soest et al. [27], are shown in Table 1.

### 2.2. Polyphenol Profile of Nano- and Micro-Encapsulated Polyphenol Extracts and Olive Leaf Pellet

Fresh leaves of the *Olea europaea* L. cv. Carolea were collected from the experimental field of the Research Centre for Olive, Fruit and Citrus Crops (CREA-OFA, Rende, Italy). Olive leaf polyphenols were extracted according to the method described by Mazzotta et al. [28], with minor operational adaptations. Briefly, the leaves were homogenized and subjected to solvent extraction using a hydroalcoholic extraction medium suitable for recovering secoiridoids and other polar phenolic compounds. The extract was then filtered to remove plant residues and clarified prior to subsequent analyses. When necessary, the extract was concentrated under reduced pressure at low temperature to avoid thermal degradation of thermolabile phenolics. The total phenolic content of the olive leaf extract was determined spectrophotometrically using the Folin–Ciocalteu reagent, according to the method reported in Fuentes et al., 2022 [29]. An aliquot of the extract was mixed with Folin–Ciocalteu reagent and an alkaline solution, and the reaction mixture was allowed to develop under controlled conditions. Absorbance was measured at 750 nm using a UV–Vis spectrophotometer. Quantification was performed by comparison with a calibration curve prepared using oleuropein as the reference standard. Results were expressed as mg of oleuropein equivalents (OE) per gram of dry encapsulated material. The qualitative and quantitative characterization of individual phenolic compounds was performed by high-performance liquid chromatography, following the method of Montedoro et al. [30]. Phenolic compounds were separated on a reversed-phase chromatographic column using a gradient elution system composed of an acidified aqueous phase and an organic solvent. Detection was carried out in the UV range at wavelengths suitable for the main olive phenolics. Individual compounds were identified by comparing retention times and spectral characteristics with those of analytical standards, when available. Quantification was performed using external calibration curves constructed with pure reference compounds. The main phenolic compounds detected in the nano-encapsulated olive leaf extract are reported in Table 2.

Nano-encapsulation of the olive leaf polyphenol extract was performed by ionic gelation using chitosan as the cationic polymer and pentasodium tripolyphosphate as the cross-linking agent, following the method described by Muzzalupo et al. [31]. The total polyphenol content of the nano-encapsulated olive leaf extract was 544.6 mg/g (OE); however, the overall mass yield of the extraction and nanoencapsulation process was not specifically determined and should be quantified in future scale-up studies. The phenolic composition is reported in Table 2.

**Table 2:** Phenolic profile of nano-encapsulated olive leaves (χ ± DS).

Compound	mg/g Encapsulated Dried Extract
Hydroxytyrosol	2.1 ± 0.7
Tyrosol	0.1 ± 0.0
4-Hydroxyphenylacetic acid	15.0 ± 1.3
Caffeic acid	0.5 ± 0.2
Ferulic acid	0.8 ± 0.3
p-Coumaric acid	0.8 ± 0.4
Oleuropein	450.1 ± 5.5
Verbascoside	21.3 ± 1.4
Ligstroside	11.4 ± 1.0
Luteolin-glycosides	42.5 ± 2.6
Chlorophyll (a + b) ^1^	1.8 ± 0.5

^1^ Chlorophyll a and chlorophyll b were determined spectrophotometrically after extraction of leaf pellet in acetone, according to the method of Lichtenthaler and Wellburn [32].

Fresh OMWW was collected during the olive oil production campaign from a three-phase extraction system. The OMWW was subjected to sequential pre-treatment steps, including sieving, centrifugation, and membrane filtration, to remove suspended solids and reduce turbidity. Maltodextrin (5% *w*/*v*) was added as a carrier agent prior to spray drying to improve encapsulation efficiency and powder stability. The OMWW–maltodextrin mixture was homogenized to ensure complete dissolution of the carrier and uniform distribution of phenolic compounds. The resulting solution was then spray-dried under controlled operating conditions to obtain a dry micro-encapsulated powder. The drying process converted the phenolic-rich liquid extract into solid microparticles suitable for feed incorporation and storage. From 1 L of OMWW, approximately 80 g of spray-dried powder were recovered, corresponding to a final production yield of about 8% *w*/*v*. The total phenolic content of the micro-encapsulated OMWW extract was determined using the Folin–Ciocalteu assay, as described above, and expressed as oleuropein equivalents. In addition, surface phenolics were quantified to estimate the fraction of phenolic compounds not fully entrapped within the carrier matrix and therefore more exposed to oxidation or degradation. The phenolic profile of the micro-encapsulated OMWW extract was determined by HPLC analysis using the same chromatographic approach described for the olive leaf extract. Identification and quantification of the main phenolic compounds were carried out using retention times, UV spectra, and calibration curves obtained from analytical standards. The phenolic composition of the micro-encapsulated OMWW extract is reported in Table 3. The total polyphenol content of the micro-encapsulated OMWW extract was 46.62 mg/g (OE).

The encapsulated products were obtained as dried materials, which facilitates handling, incorporation into feed, and short- to medium-term storage compared with liquid or non-encapsulated extracts. Encapsulation is expected to improve the protection of olive-derived polyphenols against oxidation, humidity, and degradation during storage.

Also, for the olive leaf pellet, the total phenolic content was quantified using the Folin–Ciocalteu assay, and the phenolic profile was determined by HPLC analysis (Table 4). The total polyphenol content of the olive leaf pellet was 50 mg/g (OE). Considering their low proportion in the pellet, corresponding to 1–3% of the total dry matter, the phenolic contribution from the woody fractions/branches is considered not influential on the overall phenolic profile of the pellet. Therefore, the estimated values mainly reflect the phenolic composition of the leaf fraction.

### 2.3. Production Performance

To monitor productive performance, individual animal live weights were recorded at the beginning (day 0) and at the end of the feeding trial (day 40) before slaughtering, after an overnight fast with free access to water. The average daily gain (ADG) was calculated accordingly as the linear growth rate over the 40-day experimental period. At the end of the experiment, bulls were transported to a local slaughterhouse (Montagna S.p.A.) in Corigliano-Rossano (CS) in compliance with European Union laws on Animal Welfare during transport (1/2005EC). According to the European Community regulation (1099/2009EC), animals were stunned (by captive bolt gun), exsanguinated, and dressed following commercial dressing-out procedures at the abattoir. Hot carcass weight was recorded, and dressing percentage was calculated as the percentage ratio of the hot carcass weight to the slaughter body weight. After chilling (24 h, 4 °C), *Longissimus thoracis* (LT) samples were removed from the right half of each carcass between the 11th and 13th thoracic vertebrae, vacuum packaged, and stored frozen (−20 °C) until analyses.

### 2.4. Proximate Composition

On samples of LT, proximate composition analysis was conducted according to the procedures described by the Association of Official Analytical Chemists [26]. All analyses were carried out in duplicate. Briefly, moisture and ash were calculated after drying (105 °C for 18 h) and incinerating (550 °C for 8 h) meat samples, respectively. Crude protein and total fat content were determined through the Kjeldahl copper catalyst method and the chloroform–methanol extraction described by Folch et al. [33], respectively.

### 2.5. Vitamin E and Cholesterol Content

The vitamin E (α-tocopherol) was extracted using the modified procedure of Perna et al. [34], while the cholesterol was extracted using the method of Maraschiello et al. [35]. All analyses were carried out in duplicate. Cholesterol and vitamin E were identified and quantified via HPLC (Thermo Scientific™ Vanquish™, Thermo Scientific, Waltman, MA, USA), equipped with a Hypersil GOLD™ reverse-phase column (150 mm × 4.6 mm × 3 μm; Thermo Scientific, Waltman, MA, USA) with a fluorescence detector for Vitamin E (excitation wavelength of 295 nm and emission of 325 nm) and UV-Vis detection for cholesterol at 210 nm. The mobile phase for the Vitamin E determination consisted of acetonitrile: methanol (55:45, *v*/*v*), whereas for the determination of cholesterol, a 55:45 *v*/*v* mixture of acetonitrile and isopropanol was employed, with a flow rate of 1.0 mL/min. The quantitation of muscle vitamin E and cholesterol content was based on the external standard method using a pure α-tocopherol and cholesterol standards (Sigma, St. Louis, MO, USA). Results are expressed as mg/100 g of fresh meat sample for cholesterol and as µg/g of fresh sample for vitamin E.

### 2.6. Determination of Oxidative Stability

The oxidative stability of meat was measured by evaluating the content of thiobarbituric acid reactive substances (TBARS) in muscle samples refrigerated at 4 °C by the method described by Mariutti et al. [36]. For sample quantification, a standard curve of malonaldehyde (MDA, Sigma Aldrich, St. Louis, MO, USA) was prepared by hydrolysis of tetraethoxyprpane. Results were expressed as mg of MDA per kg of meat. All samples were analysed in duplicate.

### 2.7. Fatty Acids

Fatty acid methyl esters (FAME) were prepared according to the method of Christie [37] and quantified using a Trace 1600 gas chromatograph (ThermoQuest EC Instruments, Milan, Italy) equipped with a flame ionization detector (260 °C) and a fused silica capillary column (Rt-2560, Restek, Cernusco, MI, Italy) 100 m × 0.25 mm × 0.20 μm film thickness. Helium was used as a carrier gas. The column temperature was held at 100 °C for 5 min, then raised 8 °C/min to 180 °C and maintained for 9 min, then by 1 °C/min to 230 °C and maintained for 5 min. The individual fatty acid peaks were identified by comparing the retention times with those of known mixtures of standard fatty acids (37 Component FAME MIX, Supelco, Bellefonte, PA, USA; cis/trans FAME MIX, Restek, MI, Italy; C18:2 c9,t11, NU-CHEK-PREP, INC., Elysian, MN, USA), run under the same operating conditions. The results are expressed as a percentage of the total fatty acids identified. To assess the nutritional implications, the ratio of n-6 PUFA to n-3 PUFA (n-6/n-3), the ratio of polyunsaturated fatty acids (PUFA) to saturated fatty acids (SFA) (PUFA/SFA) were calculated. The atherogenic (AI) and thrombogenic (TI) indices were computed according to Ulbricht and Southgate [38].

### 2.8. Statistical Analysis

Data on productive performance and meat quality were analyzed using the IBM SPSS Statistics software version 25 (Chicago, IL, USA) to evaluate the effect of dietary supplementation with olive-derived polyphenols. The individual animal was considered the experimental unit. Normality of data distribution was verified and confirmed using the Shapiro–Wilk test. All parameters were initially analyzed using a standard one-way analysis of variance (ANOVA) with the dietary treatment as the fixed effect. The statistical model was as follows:

Yij = μ + Ti + εij

where Yij was the dependent variable, μ was the overall mean, Ti was the fixed effect of the diet treatment (i = 3: C, T1, T2) and εij was the residual error.

Regarding the productive performance data, to ensure the robustness of the results, an analysis of covariance (ANCOVA) was further conducted, introducing the initial live weight of the animals as a covariate. This approach allowed for neutralizing any potential confounding effects caused by differences in baseline weights and isolating the true biological effect of the supplementation.

Differences between means were evaluated using the Scheffé post hoc test. Differences were considered significant at *p* < 0.05. Results are presented as mean values with standard deviation (SD) for productive performance or with standard error of the mean (SEM) for meat quality traits.

## 3. Results and Discussion

### 3.1. Rationale for Polyphenol Inclusion Levels

The inclusion levels of polyphenolic sources used in this study were established based on the dosages reported in the literature, the practical availability of olive-derived by-products, and the formulation of polyphenol extracts. The literature describes a diverse formulation, varying inclusion rates and supplementation periods, often associated with variable effects on growth performance, oxidative status, and meat quality traits [39,40,41]. Based on these references, in the present study, the dose selected for the nano-encapsulated polyphenol extract from olive leaves (T1; 40 g/head/day; total polyphenols: 544.60 mg/g OE) was defined to provide a biologically relevant supply. Under our experimental conditions, this supplementation level provided approximately 21.78 g of total polyphenols per head per day. Considering an average dry matter intake of 7 kg/day, this corresponded to approximately 3.11 g of total polyphenols per kg of DM (0.31% DM). The adoption of nano-encapsulation was intended to enhance compound stability and bioavailability, partially protecting polyphenols from ruminal degradation and potentially improving their post-ruminal absorption and systemic antioxidant effects. For the T2 group, 30 g/head/day of micro-encapsulated polyphenolic extract from OMWW (total polyphenols 46.62 mg/g OE) was included based on product availability. However, given its lower polyphenol concentration, olive leaf pellets, a by-product routinely available and already used by the farm, were also added to achieve a total polyphenol supply comparable to T1. The combined supplementation of 400 g/day of olive leaf pellets (50 mg/g total polyphenols expressed as OE) with 30 g/day of polyphenol extracts from OMWW provided approximately 21.40 g of total polyphenols per head per day, corresponding to 3.06 g/kg DM (0.30% DM). Therefore, both T1 and T2 diets delivered a comparable concentration of total polyphenols (3.0–3.1 g/kg DM), consistent with levels previously investigated in beef cattle, while allowing the comparison between a high-value polyphenol extract and a feeding strategy based on the practical use and valorization of locally available olive-derived by-products. The comparison between T1 and T2 was not intended as a simple comparison between two different polyphenol doses, but rather as a comparison between two technological and practical strategies for the valorization of olive-derived phenolics. The T1 treatment, based on nano-encapsulated olive leaf extract, represented a concentrated and high-protection delivery system, potentially able to improve phenolic stability and bioavailability. The T2 treatment, based on olive leaf pellets combined with microencapsulated olive mill wastewater extract, was designed as a more practical and scalable strategy, relying on by-products that are locally available and more easily integrated into farm-level feeding systems.

The 40-day supplementation period was selected based on previous studies reporting measurable changes in oxidative status, ruminal parameters, metabolic responses, and productive characteristics during supplementation periods ranging from 30 to 60 days [40,42,43,44]. This duration was considered sufficient to allow for physiological adaptation to the dietary treatment and the stabilization of the evaluated response variables. Additionally, this timeframe was influenced by the practical availability of the olive-derived polyphenolic extracts and the necessity to administer the established daily dose consistently throughout the experimental period.

### 3.2. In Vivo e Post Mortem Performance

Final body weight, average daily gain, hot carcass weight and yield are given in Table 5.

The dietary inclusion of olive-derived polyphenols did not affect final live weight (*p* > 0.05); however, the ADG was higher (*p* < 0.05) in the T2 group compared with the other groups. Because baseline differences in initial body weight can act as a confounding factor in growth trials, the performance data were further subjected to an analysis of covariance (ANCOVA), introducing the initial live weight as a covariate. This adjustment successfully isolated the true biological effect of the dietary treatments from any background noise caused by initial weight imbalances. The results from ANCOVA confirmed that the initial body weight had no significant linear relationship with the ADG (*p* = 0.8673 for the covariate), validating the robustness of the findings. Even after correcting for baseline variability, the superior ADG of the T2 group remained highly significant (*p* = 0.038). This statistical evidence demonstrates that the enhanced growth rate in T2 was not an artifact of compensatory growth due to a lighter starting weight, but rather a genuine physiological response to the combination of micro-encapsulated polyphenolic extract from olive mill wastewater and olive leaf pellets. These results could be related to a synergistic mechanism between the diverse phenolic profiles and delivery system characterizing the two dietary treatments (T1 and T2). In particular, T2 treatment combined olive leaf pellets (rich in oleuropein) with micro-encapsulated olive mill wastewater extract (which is predominantly rich in hydroxytyrosol), while T1 received nano-encapsulated olive leaf extract, also rich in oleuropein. Considering that oleuropein and hydroxytyrosol possess different molecular weights and amphiphilic properties, we can hypothesize different or complementary antioxidant and antimicrobial pathways. As recently highlighted by Gao et al. [45], hydroxytyrosol exhibits a wide array of biological functions in livestock production, notably improving intestinal health, protecting hepatic function, and alleviating subclinical inflammatory states. In addition, differences could be related to the different delivery systems (micro- vs. nano-encapsulated), which can have different kinetics of polyphenol release along the gastrointestinal tract.

Carcass weight and yield were also unaffected by the dietary treatment; nevertheless, the T1 group showed a tendentially lower carcass yield (−2%) than the other groups (*p* = 0.082). In agreement with the present findings, Karatosidi et al. [46] reported a hot dressing percentage of 48.91% in Podolian young bulls with a slaughter weight of 334.85 kg. Differently, a higher slaughter yield (about 55%) was observed by Marino et al. [47] in Podolian young bulls fed two forage-to-concentrate ratios, likely reflecting the greater live weight of the animals at slaughter (about 470 kg). Moreover, the dressing percentages observed in the present study were lower than those previously reported for Podolian bulls [48,49,50], which may reflect differences in rearing conditions and feeding systems. Recently, a few studies have investigated the effect of polyphenol supplementation in ruminants. In line with the results of the present study, Chiofalo et al. [51], Bionda et al. [52] and Lopreiato et al. [53] reported that olive by-products supplementation did not affect the final live weight, hot carcass, and carcass yield in beef cattle. Cimmino et al. [54] reported no adverse effects on the growth performance or carcass traits of Saanen goat kids fed a diet supplemented with polyphenols derived from olive mill wastewaters. However, to the best of our knowledge, there are no studies on Podolian bulls supplemented with polyphenols derived from olive mill wastewaters.

### 3.3. Proximate Composition, Cholesterol, Vitamin E and TBARS

Table 6 presents the chemical composition and oxidative stability, expressed as TBARS values, of the LT muscle.

Dietary supplementation with olive-derived polyphenols significantly affected muscle composition. In particular, the T2 group showed lower moisture content (*p* < 0.01) and higher protein content (*p* < 0.01) than the C and T1 groups. Moisture and protein content were similar (*p* > 0.05) between the C and T1 groups. No significant effects were observed for total lipid content (1.40–1.59%), ash, or cholesterol concentration (56.54–61.68 mg/100 g). The chemical composition observed in the present study is consistent with the findings of Giannico et al. [4], who reported similar values in the *Longissimus lumborum* muscle of Podolian young bulls. Likewise, Karatosidi et al. [46] found comparable dry matter, protein, and ash contents in the same muscle of pasture-raised Podolian young bulls, although they reported a higher lipid content (3.11%). A greater intramuscular lipid concentration (2.27 g/100 g) was also described by Cifuni et al. [50] in different muscles of Podolian cattle aged 16–18 months. Variations in lipid content among studies may be attributed to differences in livestock management systems, feeding strategies, slaughter age, and growth rates, as intramuscular fat deposition is strongly influenced by nutritional and production factors [55].

The intramuscular vitamin E (α-tocopherol), ranging from 1.44 to 2.02 µg/g, was higher in the T1 group compared with both the C and T2 groups (*p* < 0.05), while no difference was detected between the C and T2 groups (*p* > 0.05). Supplementation with olive leaves has been reported to increase tocopherol levels in meat [56]. Oleuropein, the main phenolic compound in olive by-products, exhibits strong radical-scavenging activity by stabilizing phenoxyl radicals through hydrogen bond formation, thereby limiting lipid oxidation and consequently decreasing the formation of TBARS and lipid peroxidation by-products such as malondialdehyde and 4-hydroxynonenal [57]. Beyond direct antioxidant activity, dietary polyphenols can enhance endogenous antioxidant defenses by increasing the activity of enzymes such as glutathione peroxidase, superoxide dismutase, catalase, and glutathione reductase [58], as well as by elevating glutathione synthesis via activation of the antioxidant response element and phase II enzymes [59]. Furthermore, polyphenols have health-promoting effects by modulating and interacting with various molecular pathways that enable cells to respond to both internal and external stressors. In particular, it has been demonstrated that polyphenols can regulate the nuclear factor erythroid 2-related factor 2 (Nrf2) signaling pathway, which in turn influences the expression of a wide range of downstream antioxidant response genes [45]. Regarding vitamin E, polyphenols can spare α-tocopherol by reducing tocopheroxyl radicals back to their active antioxidant form, thereby maintaining higher tissue and circulating concentrations of this vitamin. These effects reduce oxidative pressure in muscle tissues, leading to a sparing effect on vitamin E, which is less consumed as a chain-breaking antioxidant in lipid membranes. This protective effect effectively limits lipid and protein oxidation [45]. Antioxidants such as vitamin E play a significant role in preventing meat oxidation, especially long-chain PUFAs, prolonging its shelf life [21].

The results obtained in the present study with the TBARS test showed that the MDA content was markedly lower in the meat of the groups T1 and T2 than in the control (*p* < 0.01), and the amount of MDA (mg/kg) found in the T1 group was lower than that of the T2 group (*p* < 0.01). The degree of lipid oxidation in meat and processed meat products is used to provide information on the food quality and shelf-life characteristics of the meat. Some previous studies have suggested different limitations of TBARS values in beef for consumer acceptance. For example, Campo et al. [60] considered the limit threshold of TBARS values for the acceptability of oxidized beef to be approximately 2.0 mg MDA/kg; however, McKenna et al. [61] adopted 1.0 mg MDA/kg as the arbitrary threshold, while Hughes et al. [62] found that TBARS levels between 2.60 and 3.11 mg MDA/kg in long-cured beef sirloins were still acceptable to consumers. The MDA values found in the present study are well below the MDA limits indicated by the authors cited above.

### 3.4. Fatty Acid Composition

The results of the effect of dietary inclusion of olive-derived polyphenols on the fatty acid composition of the LT muscle are presented in Table 7. As regards the fatty acid profile, SFAs were predominant (47.50–50.95%), followed by MUFAs (32.22–36.82%) and PUFAs (15.68–18.99%). Similar results are reported in the literature for different muscles of Podolian cattle [46,47,50]. The high SFA content of ruminant meat is largely due to ruminal biohydrogenation of dietary unsaturated fatty acids by microorganisms, which limits their transfer to the small intestine and results in enrichment of absorbed SFA in body tissues [63].

The dietary inclusion of olive-derived polyphenols did not affect the total SFAs and PUFAs, while MUFAs tended to be lower (*p* = 0.057) in treated groups (T1 and T2) as compared to the C group. This trend was mainly attributable to the tendentially lower percentage of oleic acid (C18:1 c9; *p* = 0.076), palmitoleic acid (C16:1; *p* < 0.01) and heptadecenoic acid (C17:1; *p* < 0.01) in treated groups. In contrast, a study on dietary olive cake supplementation in Limousin young bulls [51] reported an increase in oleic acid and other trans isomers of oleic (C18:1 t11 and t10) in supplemented groups compared with the control. These differences suggest that the effect of olive by-products on MUFA content may be more pronounced when the whole matrix (including residual oil) is provided, compared with supplementation using isolated polyphenolic extracts. Regarding individual PUFAs, dietary inclusion of olive-derived polyphenols did not affect the content of linoleic acid (C18:2 n-6), the main precursor of n-6 PUFAs. However, meat from the T2 group exhibited a significantly higher proportion of CLA 9c,11t (rumenic acid, RA; *p* < 0.05) compared with the T1 group, but similar to those observed in the C group (*p* > 0.05). This result may be explained by the ability of specific phenolic compounds to modulate rumen microbial activity. Several in vitro studies have shown that polyphenols can partially inhibit ruminal biohydrogenation of fatty acids by affecting key microbial populations involved in this process. In particular, plants rich in polyphenols appear to reduce the extent of PUFA biohydrogenation in the rumen, thereby increasing the flow of these intermediates to the intestine and promoting greater tissue deposition of CLA, especially rumenic acid [54]. Significant differences were observed for α-linolenic acid (C18:3 n-3), the precursor of n-3 PUFAs, which was higher (*p* < 0.01) in the T2 group as compared to T1 and C groups. Similarly, the long-chain n-3 PUFAs eicosapentaenoic acid (EPA, C20:5 n-3) and docosapentaenoic acid (DPA, C22:5 n-3) were affected by inclusion of olive-derived polyphenols: EPA was higher in T2 compared with the C group (*p* < 0.05) and DPA, which was tendentially higher (DPA, *p* = 0.082) in the T2 group compared with the other groups. Consequently, the total n-3 PUFA content was higher (*p* < 0.05) in the T2 group than in the C group, with the T1 group showing an intermediate value (*p* > 0.05). Therefore, the observed enrichment of n-3 PUFAs in the treated groups (T1 and T2 groups) is consistent with the documented ability of polyphenols to modulate rumen microbial ecology and inhibit key steps of fatty acid biohydrogenation, ultimately leading to a more favourable fatty acid profile in ruminant meat [64,65,66,67].

About nutritional indices, an improved n-6/n-3 ratio was observed in both T1 and T2 groups compared with the control (*p* < 0.01), and T2 compared with T1 (*p* < 0.05). This improvement is nutritionally relevant, as a lower n-6/n-3 ratio is widely associated with beneficial effects on human health, particularly in reducing the risk of cardiovascular diseases and inflammatory disorders [68]. The observed modulation of the fatty acid profile may be related to the already mentioned ability of dietary polyphenols to influence ruminal biohydrogenation processes, thereby favoring the preservation and deposition of n-3 PUFAs in ruminant tissues [64]. The n-6/n-3 ratio deviated from the ideal value of 1 in all groups, but remained below the recommended maximum of 4 in T1 and T2. Only the C group exceeded this limit, showing a higher and less nutritionally favorable ratio compared with both supplemented groups. The PUFA/SFA ratio recorded in the present study was relatively low, ranging from 0.33 to 0.40, which is slightly below the recommended threshold of >0.4 for a nutritionally favorable lipid profile [69]. The atherogenic (AI) and thrombogenic (TI) indices provide an estimate of the potential pro- or anti-atherogenic and thrombogenic effects of the fatty acid composition. The AI and TI values obtained here were comparable to those reported by Marino et al. [47]. In general, lower AI and TI values are considered desirable to minimize cardiovascular risk [70].

### 3.5. Strengths and Limitations of the Study

A key strength of this study is its innovative application of nano- and micro-encapsulation technologies for the targeted delivery of olive-derived polyphenols. This approach was intended to potentially enhance the health and growth of animals, as well as improve the quality of the products they produce. Additionally, the research demonstrates a practical and eco-friendly methodology that aligns with circular economy principles and promotes sustainable livestock production in representative commercial settings. However, these commercial settings contributed to one of the main weaknesses of the study: the relatively small number of animals involved. Indeed, identifying uniform cohorts of autochthonous cattle that meet the required experimental criteria within small- to medium-sized farms in the inner and marginal areas of Southern Italy poses significant logistical challenges. Nevertheless, these findings offer valuable, practical insights that bridge the gap between strictly controlled laboratory environments and real livestock management.

From a practical and industrial perspective, the large-scale commercial application of encapsulated olive-derived polyphenols in ruminant feeds will depend on the balance between biological efficacy, production cost, process complexity, and compatibility with existing feed-manufacturing systems. The spray-drying-based microencapsulation of OMWW appears more suitable for short-term scale-up, as it uses a liquid agro-industrial by-product and generates a dry powder that can be easily incorporated into compound feeds or supplements. In contrast, nanoencapsulation by ionic gelation may provide greater protection and bioavailability of polyphenols, but it is currently more technically demanding and likely more expensive for large-scale feed production. Therefore, while the present results support the principle that encapsulated olive polyphenols can improve oxidative stability and selected meat-quality traits, commercial viability will require further optimization of production yield, ingredient cost, storage stability, and dose efficiency. In the short term, strategies based on locally available olive by-products, such as olive leaf pellets combined with simpler microencapsulated extracts, may represent the most feasible route for practical application in ruminant feeding systems.

## 4. Conclusions

The integration of olive-derived by-products into ruminant feeding strategies aligns with the principles of a circular economy by repurposing agricultural residues. While a comprehensive evaluation of the long-term environmental impact of these improved dietary treatments requires a lifecycle assessment, using these by-products in animal nutrition provides an effective approach to waste reduction. This study demonstrates that dietary supplementation with olive-derived encapsulated polyphenols for 40 days represents a viable strategy for improving the nutritional characteristics of meat from Podolian young bulls. While this duration allowed for physiological adaptation, caution is required when interpreting long-term performance due to the small sample size. Regarding productive performance, the combined inclusion of olive leaf pellets and micro-encapsulated polyphenols from olive mill wastewater (T2) showed a positive trend in average daily gain. In terms of nutritional composition, T2 increased the protein content, while nano-encapsulated polyphenol extracts from olive leaf (T1) elevated the intramuscular vitamin E level. Both supplemented groups exhibited markedly lower malondialdehyde concentrations, confirming a significant improvement in oxidative stability. These results highlight the potential of encapsulated olive by-products as functional feed ingredients to enhance the quality and shelf-life of meat. Dietary polyphenols improved the meat fatty acid profile: enhanced the n-3 PUFA, reduced n-6/n-3 ratio in both treated groups, with T2 achieving the lowest value and therefore most favorable. Other nutritional indices were not significantly affected. Future research should involve larger animal cohorts to validate these growth performance trends and the sensory profile of the meat. Additionally, advanced omics approaches should be used to investigate the underlying mechanisms of ruminal and metabolic modulation.

## Figures and Tables

**Table 1:** Ingredients and chemical composition of the farm diet and pasture.

Item ^1^	Olive Leaf Pellet	Meadow Hay ^2^	Pasture	Mixed Coarse Meal (Wheat, Barley, and Fava Bean)
Dry matter, %	88.00	91.78	34.50	89.33
Crude protein, % of DM	9.01	4.06	8.64	12.52
Ether extract, % of DM	4.98	1.66	2.91	4.37
Crude fiber, % of DM	18.96	38.49	29.80	10.08
Neutral Detergent Fiber, % of DM	40.10	69.30	61.90	34.88
Acid Detergent Fiber, % of DM	35.05	45.39	38.40	12.34
Acid Detergent Lignin, % of DM	18.10	7.67	9.20	1.45
Ash, % of DM	5.02	5.78	7.70	2.83
Fatty acids (% on total fatty acids)				
Saturated fatty acids		27.25	37.21	20.95
Monounsaturated fatty acids		39.27	8.74	25.41
Polyunsaturated fatty acids		33.47	54.05	53.64

^1^ DM = dry matter. ^2^ The hay refers to mixed-species meadow hay derived from permanent multispecies grasslands, composed of native Mediterranean grasses (*Poaceae*), such as *Lolium* spp., *Festuca* spp., *Dactylis glomerata*, and *Poa pratensis*, together with legumes (*Fabaceae*), mainly *Trifolium* spp., *Medicago sativa*, and *Lotus corniculatus*, as well as other local forbs typical of the hilly areas of Southern Italy.

**Table 3:** Phenolic profile of olive mill wastewater (OMWW) filtered and dried microparticles of olive mill wastewater (χ ± DS).

Compound	Filtered OMWW mg/L	Dried OMWW Microparticles mg/kg Powder
Hydroxytyrosol	3451.0 ± 39.8	1405.0 ± 16.2
Tyrosol	439.0 ± 5.1	127.0 ± 1.5
Vanillic acid	191.0 ± 2.1	87.0 ± 1.0
4-Hydroxyphenylacetic acid	67.0 ± 0.8	21.0 ± 0.6
Caffeic acid	3.0 ± 0.1	1.4 ± 0.0
Ferulic acid	2.0 ± 0.0	1.1 ± 0.0
p-Coumaric acid	1.0 ± 0.0	0.6 ± 0.0
Oleuropein	Traces	n.d.
Verbascoside	Traces	n.d.
Ligstroside	Traces	n.d.
Luteolin-glycosides	Traces	n.d.

n.d. = not detected.

**Table 4:** Phenolic composition of olive leaf pellets from ‘Carolea’ pellet, 97–99% leaves (χ ± DS).

Phenolic Compound	mg/kg dw
Hydroxytyrosol	2.08 ± 0.09
Tyrosol	0.11 ± 0.02
4-hydrossyphenylacetic acid	14.5 ± 1.10
Caffeic acid	0.42 ± 0.15
Ferulic acid	0.70 ± 0.21
Coumaric acid	0.14 ± 0.08
Oleuropein and derivatives	392.52 ± 15.90
Verbascoside	16.54 ± 0.90
Luteolin-O-glucoside	36.42 ± 1.80
Chlorophyll (a + b) ^1^	1.18 ± 0.40

^1^ Chlorophyll a and chlorophyll b were determined spectrophotometrically after extraction of leaf pellet in acetone, according to the method of Lichtenthaler and Wellburn [32].

**Table 5:** In vivo and post-mortem performance (χ ± DS) of Podolian young bulls fed diets supplemented with olive-derived polyphenols.

	Group ^1^			*p*-Value
	C	T1	T2	
Initial live weight, kg	294 ± 29.23	317 ± 21.34	279 ± 42.25	0.247
Final live weight, kg	330 ± 32.22	348 ± 16.99	331 ± 44.29	0.617
ADG, Kg	0.900 ± 0.147 ^b^	0.790 ± 0.233 ^b^	1.300 ± 0.087 ^a^	0.011
Hot Carcass weight, kg	161 ± 15.55	161 ± 10.03	160 ± 19.67	0.990
Hot carcass yield, %	48.9 ± 0.23	46.2 ± 0.97	48.5 ± 3.20	0.082

^1^ C = group fed with standard diet; T1 = group fed with standard diet supplemented with 40 g/day/head of nano-encapsulated polyphenolic extract from olive leaves; T2 = group fed with standard diet supplemented with 400 g/day/head of olive leaf pellets and 30 g/day/head of micro-encapsulated polyphenolic extract from olive mill wastewater. ^a,b^ Means within a row with different lowercase superscripts differ (*p* < 0.05).

**Table 6:** Chemical composition, cholesterol, vitamins E and TBARs of Longissimus thoracis of Podolian young bulls fed diets supplemented with olive-derived polyphenols.

	Group ^1^			SEM	*p*-Value
	C	T1	T2		
Moisture, %	76.22 ^A^	76.14 ^A^	74.98 ^B^	0.150	0.000
Protein, %	21.34 ^B^	21.52 ^B^	22.54 ^A^	0.138	0.000
Lipids, %	1.59	1.40	1.42	0.059	0.371
Ash, %	1.07	1.06	1.06	0.012	0.883
Cholesterol, mg/100 g	59.07	61.68	56.54	1.253	0.270
Vitamin E, µg/g	1.44 ^b^	2.02 ^a^	1.56 ^b^	0.094	0.013
TBARS, mg of MDA/100 g	0.328 ^A^	0.119 ^C^	0.215 ^B^	0.029	0.004

^1^ C = group fed with standard diet; T1 = group fed with standard diet supplemented with 40 g/day/head of nano-encapsulated polyphenolic extract from olive leaves; T2 = group fed with standard diet supplemented with 400 g/day/head of olive leaf pellets and 30 g/day/head of micro-encapsulated polyphenolic extract from olive mill wastewater. ^a,b^ Means within a row with different lowercase superscripts differ (*p* < 0.05); ^A–C^ means within a row with different uppercase superscripts differ (*p* < 0.01).

**Table 7:** Fatty acid composition of Longissimus thoracis from Podolian young bulls fed diets supplemented with olive-derived polyphenols.

	Group ^1^			SEM	*p*-Value
Fatty Acid, % on Total Fatty Acids	C	T1	T2		
C14:0	2.04	1.97	2.29	0.108	0.502
C15:0	0.59	0.74	0.76	0.049	0.313
C16:0	24.26	25.44	24.43	0.440	0.496
C17:0	1.10	1.23	1.20	0.049	0.565
C18:0	19.15	21.23	19.49	0.586	0.284
C20:0	0.19	0.17	0.20	0.009	0.554
C22:0	0.17	0.17	0.18	0.007	0.672
C14:1	0.22	0.16	0.18	0.017	0.262
C16:1	2.96 ^A^	2.34 ^B^	2.49 ^B^	0.094	0.004
C17:1	0.55 ^A^	0.49 ^B^	0.46 ^B^	0.011	0.001
C18:1 c9	30.59	26.55	26.33	0.880	0.076
C18:1 c11	0.99	0.87	1.05	0.046	0.302
C18:1 t11	1.38	1.73	1.85	0.129	0.347
C20:1	0.13	0.08	0.08	0.011	0.084
C18:2 n6	9.51	9.70	10.17	0.434	0.859
C18:2 c9, t11	0.39 ^ab^	0.35 ^b^	0.44 ^a^	0.015	0.046
C18:2 t9, t12	0.14 ^b^	0.12 ^b^	0.17 ^a^	0.007	0.014
C18:3 n3	1.45 ^b^	1.44 ^b^	2.27 ^a^	0.128	0.005
C18:3 n6	0.04	0.06	0.05	0.003	0.106
C20:2 n6	0.10	0.11	0.12	0.007	0.791
C20:3 n6	0.54	0.59	0.63	0.043	0.740
C20:4 n6	2.14	2.42	2.54	0.191	0.718
C20:5 n3	0.33 ^b^	0.62 ^ab^	0.76 ^a^	0.070	0.034
C22:2 n6	0.11 ^b^	0.12 ^b^	0.18 ^a^	0.011	0.021
C22:5 n3	0.81	1.20	1.51	0.125	0.082
C22:6 n3	0.12	0.10	0.14	0.011	0.252
SFA	47.50	50.95	48.56	1.051	0.380
MUFA	36.82	32.22	32.45	0.922	0.057
PUFA	15.68	16.82	18.99	0.936	0.420
**Nutritional indices**					
PUFA/SFA	0.33	0.33	0.40	0.036	0.392
n-6 PUFA	12.48	13.00	13.69	0.661	0.814
n-3 PUFA	2.70 ^b^	3.35 ^ab^	4.68 ^a^	0.304	0.025
n-6/n-3	4.62 ^A^	3.88 ^B^	2.93 ^C^	0.226	0.001
AI	0.63	0.70	0.67	0.029	0.684
IT	1.41	1.51	1.27	0.071	0.436

^1^ C = group fed with standard diet; T1 = group fed with standard diet supplemented with 40 g/day/head of nano-encapsulated polyphenolic extract from olive leaves; T2 = group fed with standard diet supplemented with 400 g/day/head of olive leaf pellets and 30 g/day/head of micro-encapsulated polyphenolic extract from olive mill wastewater. l SFA = saturated fatty acids; MUFA = monounsaturated fatty acids; PUFA = polyunsaturated fatty acids; AI = atherogenic index; IT = thrombogenic index; SEM = Standard Error Mean. ^a,b^ Means within a row with different lowercase superscripts differ (*p* < 0.05); ^A–C^ means within a row with different uppercase superscripts differ (*p* < 0.01).

## Data Availability

The data that support the findings of this study are available from the corresponding author upon reasonable request.

## Supplementary Materials

The following supporting information can be downloaded at: https://www.mdpi.com/article/10.3390/ani16121791/s1, Table S1. Experimental design, dietary treatments, and supplementation doses.

## Abbreviations

The following abbreviations are used in this manuscript:

PE	Polyphenol extracts
OL	Olive leaves
OMWW	Olive mill wastewater
DM	Dry matter
ADG	Average daily gain
OE	Oleuropein equivalents
LT	Longissimus thoracis
TBARS	Thiobarbituric Acid Reactive Substances
MDA	Malonaldehyde
FAME	Fatty acids methyl esters
SFA	Saturated fatty acids
MUFA	Monounsaturated fatty acids
PUFA	Polyunsaturated fatty acids
AI	Atherogenic index
TI	Thrombogenic index
