# Supplementary material for: Dietary Encapsulated Olive-Derived Polyphenols: Productive Performance and Meat Quality in Podolian Young Bulls
Source: Animals (Basel). 2026 Jun 10;16(12):1791. doi: 10.3390/ani16121791 (PMC13295315; doi:10.3390/ani16121791)
Supplement: Supplementary file 1 [file animals-16-01791-s001.zip › animals-4319323-supplementary.pdf]

Supplementary Table S1. Experimental design, dietary treatments, and supplementation doses.

Group	Basal Diet	Supplementation Dose
**Control (C)**	Concentrate (4.0kg/day; based on durum wheat flour, barley, and faba bean) + meadow hay (3.0kg/day), supplied at approximately 2% of the initial live weight	None
**T1**		40g/day/head of nano-encapsulated polyphenolic extracts from olive leaves (OL)
**T2**		400g/day/head of olive leaf pellets + 30g/day/head of micro-encapsulated polyphenolic extract from olive mill wastewater (OMWW)
